# TiO_2_ Nanoparticles Enabling Photocatalytic Desulfurization for C–C Coupling Reaction Using Visible Light

**DOI:** 10.1021/acsnanoscienceau.6c00020

**Published:** 2026-02-27

**Authors:** Shea Stewart, Matas Simukaitis, H. Christopher Fry, Chengjun Sun, Yugang Sun

**Affiliations:** † Department of Chemistry, 6558 Temple University, 1901 North 13th Street, Philadelphia, Pennsylvania 19122, United States; ‡ Center for Nanoscale Materials, 1291 Argonne National Laboratory, 9700 South Cass Avenue, Lemont, Illinois 60439, United States; § X-ray Science Division, Advanced Photon Source, Argonne National Laboratory, 9700 South Cass Avenue, Lemont, Illinois 60439, United States

**Keywords:** photocatalytic thiol desulfurization, reactive carbon radicals, addition of carbon radical to vinyl groups, heterogeneous photocatalysis, surface complex absorbing visible light

## Abstract

We uncover a surface-mediated mechanism for visible-light-driven photocatalytic desulfurization of thiols on titanium dioxide (TiO_2_) nanoparticles under LED illumination (>420 nm). Combined diffuse reflectance, infrared, and cryogenic electron paramagnetic resonance spectroscopy verify the formation of a surface thiol–Ti charge-transfer complex that introduces visible-light absorption into an otherwise UV-active reaction system. Selective photoexcitation of this surface complex drives interfacial charge transfer, directly activating the adsorbed thiol and inducing C–S bond scission without the band gap excitation of TiO_2_. The resulting carbon-centered radical intermediates are intercepted by styrene to form C–C coupling products. These results uncover a distinct surface-mediated photocatalytic mechanism in which charge-transfer complex formation governs both light absorption and chemical activation, providing a mechanistic framework for extending visible-light reactivity to wide-bandgap metal oxide photocatalysts.

## Graphical abstract

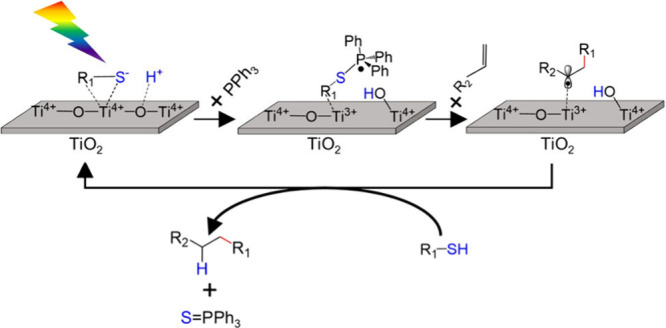

Photocatalytic desulfurization of thiols with phosphines is a promising approach for accessing reactive carbon-centered radicals. Using visible light has recently garnered strong attention to drive the desulfurization process due to its advantages compared to ultraviolet (UV) light.^1–3^ The accessibility of carbon radicals is particularly useful for forming new C–C bonds to alkenes in a Giese-like fashion. For example, an application of such C–C coupling reactions is to functionalize cysteine −SH handles on peptide chains, allowing for late-stage site-selective modification of these chains.^4^ The methods to achieve visible-light-driven desulfurization of thiols usually rely on homogeneous catalytic systems using precious metal-centered molecular photocatalysts, with some examples like Ir,^5^ Ru,^6^ and Au.^7^ Other homogeneous photocatalysts used for this purpose include organic dye molecules^8^ and light-absorbing aggregates^9^ or molecules^10^ formed in situ. It has also been reported in the literature that near-UV light can drive desulfurization for solutions containing only thiol and phosphine.^11^ We have also reported that a possible triphenylphosphine (TPP)/triphenylphosphine oxide (TPPO) aggregate is responsible for absorbing near-UV light to activate the reaction reagents.^12^ On the other hand, no heterogeneous catalysts have been employed for photocatalytic desulfurization of thiols, although heterogeneous photocatalysis is widely used in a wide range of chemical synthesis due to the unique advantages of heterogeneous catalysts (e.g., low cost, easy recovery, strong light absorption, etc.). We have studied the use of titanium nitride (TiN) nanoparticles, which strongly absorb visible light due to surface plasmon resonances, to drive desulfurization of methyl thioglycolate by TPP under illumination of white LED light with wavelengths of >420 nm.^13^ Despite the strong light absorption power in the TiN nanoparticles, the absorbed light is essentially converted to heat through the photothermal effect to elevate the temperature of the reaction solution, accelerating the reaction kinetics. When the TiN nanoparticles are partially oxidized to form surface layers of titanium oxides, the acceleration of reaction kinetics becomes more significant, even though overall light absorption drops. Such a controversial dependence of reaction kinetics on light absorption intrigues us to find the role of TiO_2_, which does not absorb visible light of >420 nm, in promoting the desulfurization reaction. Herein, we report the use of commercial pure TiO_2_ nanoparticles as a heterogeneous photocatalyst for the desulfurization of thiols to produce reactive carbon radicals, which can be captured by styrene derivatives to form new C–C coupling products. A suite of comprehensive spectroscopic studies has been employed to investigate the complexes of thiol molecules adsorbed on TiO_2_ surfaces formed in the dark and their transformation under photoillumination. The results reveal the importance of forming surface complexes in absorbing visible light to activate the reaction reagents and enable the following desulfurization and C–C coupling reactions.

We have used P25 TiO_2_ nanoparticles to facilitate the reductive C–C functionalization of arylalkenes with the intermediates derived from visible-light-driven thiol desulfurization in the presence of TTP. A typical example is the coupling of methyl thioglycolate (MTG, **1**) and styrene (STY, **2**) to yield methyl 4-phenylbutyrate (**3a**) (Scheme 1). Table 1 shows the yield of **3a** under various reaction conditions. Entries 1–5 explore the effect of reagent ratios, indicating that excessive MTG and TPP are needed to achieve desirable yields of **3a**, which is calculated against the amount of the limiting reagent, **2**. Two equivalents of both MTG and TPP yield the highest amount of **3a** (Entry 4), indicating that higher coverage of MTG on TiO_2_ nanoparticle originating from higher concentration of MTG favors the photocatalytic reaction kinetics. The recipe in Entry 5 is typically used, as the reduction of 0.5 equiv of TPP does not significantly lower the yield. The excess of MTG and TPP compensates for the side reactions resulting from the oxidation of the reagents under an air atmosphere and the direct desulfurization of MTG. The absence of product **3a** from the reaction of Entry 6 highlights the crucial role of visible-light illumination in enabling the C–C coupling reaction. Although heating can accelerate the reaction, it cannot initiate the reaction. Adding 0.8 mmol of (2,2,6,6-tetramethylpiperidin-1-yl)­oxyl (TEMPO) to the reaction solution suppresses the production of **3a** (Entry 7), implying the involvement of radicals in the photocatalytic C–C coupling reaction. When the P25 TiO_2_ nanoparticles are replaced with pure-phase anatase TiO_2_ nanoparticles with smaller sizes (5 nm), the yield of **3a** exhibits only a slight increase (Entry 8). This similarity in reactivity excludes the possibility that the narrower band gap rutile TiO_2_ is responsible for the visible-light photocatalytic activity. Switching MTG to its corresponding disulfide leads to no yield of **3a** (entry 9), indicating that the disulfide impurity present in MTG is not responsible for the observed C–C coupling reaction.

**Scheme 1:**
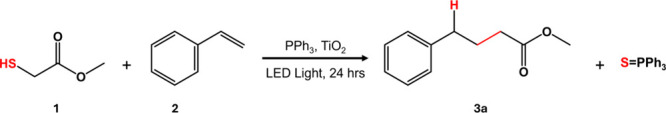
Exemplar C–C Coupling Reaction via Photocatalytic Desulfurization in the Presence of TiO_2_ Nanoparticles under Illumination of Visible Light with Wavelengths of >420 nm Produced by an LED Lamp^a^

a The reaction was performed under ambient atmosphere. The red stick in **3a** indicates the newly formed C–C bond.

**Table 1:** Yield of Methyl 4-Phenylbutyrate (**3a**) under Different Reaction Conditions for the Reaction Shown in Scheme 1

Entry	MTG (**1**): STY (**2**): TPP^a^	Yield of **3a** ^b^	Condition^c^
1	1:1:1	40%	Standard
2	2:1:1	57%	Standard
3	1:1:2	45%	Standard
4	2:1:2	70%	Standard
5	2:1:1.5	67%	Standard
6	2:1:1.5	0%	No light, 60 °C
7	2:1:1.5	0%	Addition of 1 mmol TEMPO
8	2:1:1.5	73%	5 nm anatase TiO_2_ nanoparticles
9	1:1:1.5	0%	Replacing **1** with disulfide

a 1 equiv of reagent is equal to 0.4 mmol, and the amount of TiO_2_ nanoparticles is 5 mg.

b The yield of **3a** is calculated against the amount of **2**.

c Standard condition refers to that used in the reaction presented in Scheme 1, including 5 mg P25 TiO_2_ nanoparticles, illumination with LED light with wavelength >420 nm, and room temperature. The nonstandard conditions only highlight the changes to the standard condition.

The photocatalytic C–C coupling via desulfurization using TiO_2_ nanoparticles as the visible-light photocatalyst has the potential to be extended to other reactant substrates. Figure 1 summarizes the reaction results using different reactants in which different styrene derivatives are coupled with either MTG or thioacetic acid (TAA). The intermediate derived from the desulfurization of MTG can always couple with styrene (i.e., **3a**-**3g**), although the yield varies depending on the structure of the styrene. Coupling MTG with 2-bromostyrene gave a lower yield of **3d**. The inductive withdrawing effect of Br and the increased steric bulkiness imparted by Br may hinder the approach of alkene molecules to the desulfurization intermediates, thereby slowing the reaction kinetics. The low yields of **3f** and **3g** indicate that the kinetics of the C–C coupling is severely hindered when considering alkenes bearing substituents at the reaction site, which may be explained in terms of steric clashes of the substituent with the TiO_2_ surface. Extending the reaction time from 24 to 48 h increases the yield of **3f** from 20% to 38%. Elevating the reaction temperature to 60 °C increases the yield of **3f** to 88% while maintaining the same reaction time. The low yields reported in Figure 1 are not an issue, as the yield of C–C coupling products can be increased by elongating the reaction time or performing the reactions at higher temperatures. In contrast, the yield of **3e** is as high as 55% under standard conditions, although 1-indene is structurally similar to cis-β-methylstyrene (i.e., precursors for **3f** and **3g**). The lack of free rotation of the bonds due to the rigidity of the five-membered ring might be responsible for the higher reactivity of 1-indene, while the free rotation at the reaction site of cis-β-methylstyrene may hinder the reactivity.

**Figure 1:**
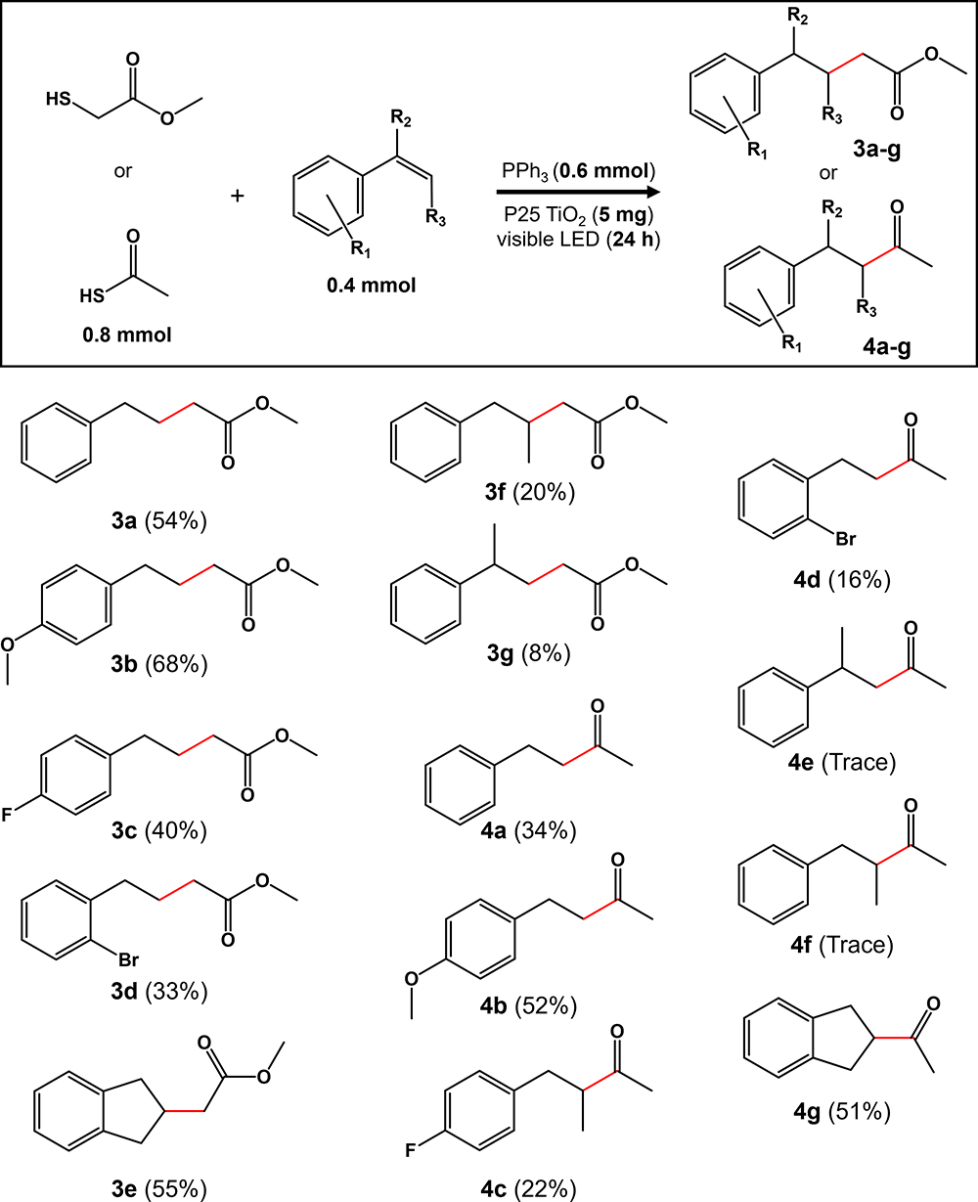
Reactant substrate scope for C–C coupling using the same conditions as those of Entry 5 shown in Table 1. Yields, i.e., the values in parentheses, were determined by examining the crude reaction mixtures via ^1^H NMR, with quantification made in reference to trans-cinnamaldehyde.

When using TAA to provide an intermediate radical via photocatalytic desulfurization, the reactivity for C–C coupling with styrene derivatives exhibits a trend similar to that of MTG (**4a**–**g** versus **3a**–**g**, Figure 1). Electron-rich styrene better couples with the intermediate radical as long as there is a lack of steric hindrance at the alkene site. The higher yield of **4a** than **4c** (34% versus 22%) indicates electrophilicity of the intermediate acyl radical derived from desulfurization of thiol.^14–16^ The yield of the C–C coupling product (i.e., **4b**) is significantly higher when F at the para position is substituted with an electron-donating methoxy group (CH_3_O−). An additional experiment where both 4-fluoro and 4-methoxystyrene of the same amount are added to a reaction solution shows a higher amount of C–C coupling product arising from the 4-methoxystyrene (Figure S1), further confirming the electrophilicity of the acyl radical.

The TiO_2_ nanoparticles can be recycled to run additional reactions without a significant reduction in their photocatalytic activity. Figure 2A compares the yield of **3a** for five consecutive reactions of Scheme 1 using the same TiO_2_ catalyst recycled through centrifugation, showing only a slight decrease in yield that might be due to the loss of TiO_2_ nanoparticles during the recovery process. Crystalline structure of the TiO_2_ nanoparticles remains essentially unchanged after the use in photocatalytic reaction, as reflected by the very similar XRD patterns presented in Figure 2B. Although the recoverability and stability of the TiO_2_ nanoparticles make them consistent with the characteristics of a photocatalyst, the wide bandgap of the TiO_2_ nanoparticles cannot allow them to absorb the visible LED light with wavelengths of >420 nm. Since the TiO_2_ nanoparticles themselves are not responsive to visible light, it is necessary to determine the actual species that can absorb visible light to drive the desulfurization of thiols and the consecutive C–C reactions.

**Figure 2:**
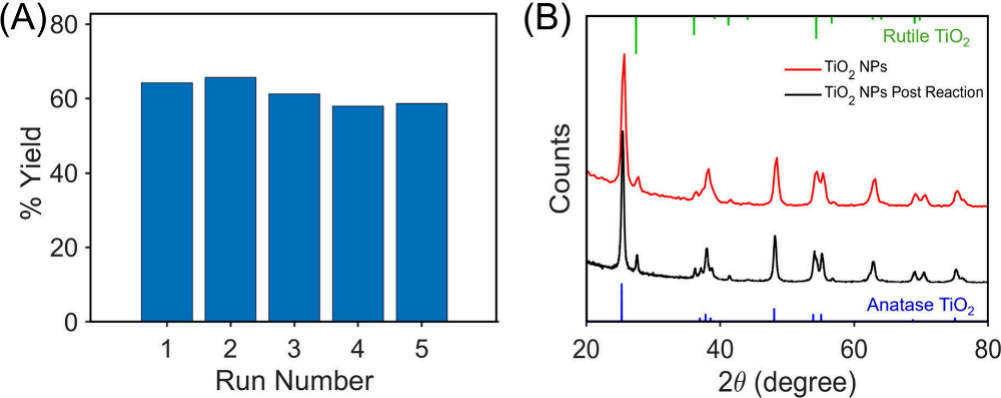
Evaluation of the stability of the P25 TiO_2_ nanoparticles used for photocatalytic reactions. (A) Yields of product **3a** from the reaction of Scheme 1 under the same conditions using the same batch of TiO_2_ nanoparticles, which were recovered after each reaction. (B) XRD patterns of the same batch of the TiO_2_ nanoparticles before (red curve) and after (black curve) reaction. The standard XRD patterns of anatase and rutile phases of TiO_2_ (i.e., blue sticks and green sticks) are also presented as a reference.

Optical absorption spectroscopy is useful for characterizing the ability of interesting species to absorb light at specific wavelengths. Due to the influence of nanoparticle scattering, diffuse reflectance spectroscopy (DRS) is used to characterize nanoparticles incorporated with reactants, thereby achieving the actual optical absorbance. Incubating TiO_2_ nanoparticles dispersed in an acetonitrile solution of MTG results in a dispersion with a light-yellow color (inset, Figure 3A), indicating that the adsorption of MTG on the TiO_2_ surface forms surface species capable of absorbing blue light. The DRS spectrum of the TiO_2_ nanoparticles covered with MTG exhibits a strong peak centered at 420 nm (solid curve, Figure 3A), corresponding to the formation of a charge-transfer complex (CT-complex) between MTG and the surface TiO_2_ lattices. The MTG-TiO_2_ CT-complex is responsible for absorbing visible LED light to drive the photocatalytic reactions described in the previous section (see Scheme 1, Table 1, and Figure 1). The MTG-modified TiO_2_ nanoparticles exhibit infrared (IR) absorption at 1560 cm^–1^ and 1400 cm^–1^ (solid red curve, Figure 3B), indicating the chelate coordination between MTG molecules and surface-Ti atoms.^17^ Both S and carbonyl O of an MTG molecule coordinate with the same surface Ti atom in the chelation structure (Figure 3C). This is corroborated by the absence of a DRS peak around 420 nm for the TiO_2_ nanoparticles modified with benzyl mercaptan (Supporting Information, Figure S2), as benzyl mercaptan molecules lack oxygen and therefore cannot form a chelation coordination. The MTG-modified TiO_2_ nanoparticles are subject to a change in both DRS and IR spectra after being illuminated with the LED lamp for 15 min at room temperature. The strong DRS peak at 420 nm disappears after photoillumination, while absorbance at shorter wavelengths increases dramatically and broadband peakless absorption at wavelengths of >400 nm also emerges (dashed curve, Figure 3A). Such spectral changes indicate the occurrence of a reaction of surface-adsorbed MTG under photoillumination. The disappearance of the 420 nm peak indicates that MTG molecules are converted to other molecules under photoillumination. The optical absorption at wavelengths below 350 nm implies the formation of a disulfide. The ^1^H NMR studies support the conversion of MTG to the corresponding disulfide. Washing MTG-modified TiO_2_ nanoparticles with CDCl_3_ before and after photoillumination yields solutions that are observed to contain essentially MTG and only disulfide, respectively (Supporting Information, Figure S3). From these observations, it is reasonable to argue that the adsorbed MTG molecules under photoillumination are transformed to thiyl radicals, which are more reactive and couple to form a stable disulfide. The conversion of MTG to disulfide is an oxidation process, and correspondingly, surface coordinated Ti­(IV) should be reduced to Ti­(III) to balance the charges during the photoreaction. The reduction of Ti^4+^ to Ti^3+^ is reflected from the X-ray absorption near edge structure (XANES) spectroscopy, which shows the shift of Ti K-edge to a lower energy after photoillumination (Supporting Information, Figure S4). Such a shift indicates the lowering of the oxidation state of titanium in the photoilluminated MTG-modified TiO_2_ nanoparticles. The reduction of a Ti­(IV) atom breaks a Ti–O bond to create an oxygen vacancy site on the nanoparticle surface, and the O atom binds to the proton dissociated from the MTG molecule. The surface oxygen vacancies and Ti­(III) atoms extend the optical absorption of the TiO_2_ nanoparticles to the visible region, which is consistent with broadband absorption beyond 400 nm. The IR transmission spectrum of the photoilluminated nanoparticles exhibits similar peaks to the sample before photoillumination, except for a redshift to lower energies (dashed red curve, Figure 3B). The remaining peak profile indicates that the disulfide molecules are still likely to maintain the chelating adsorption geometry with surface Ti atoms. The redshift of the C = O stretching peak from 1735 cm^–1^ to 1715 cm^–1^ implies that the carbonyl O atoms in disulfide bind stronger to the surface Ti atoms than MTG. The involvement of redox processes and radicals in the light-driven conversion of MTG to disulfide is confirmed through cryo-electron paramagnetic resonance (EPR) characterizations. The EPR spectrum of the MTG-modified TiO_2_ nanoparticles after photoillumination confirms the presence of Ti^3+^ centers (i.e., lattice-Ti trapped electrons or Ti­(III) atoms),^18^ which correspond to the peak at g = 1.991 and two valleys at 1.985 and 1.959 (Figure 3D). The strong valley signal at ∼2.000 originates from the F centers, i.e., oxygen vacancies occupied by single electrons. Coexistence of Ti^3+^ and oxygen vacancies in reduced TiO_2_ is common to maintain charge balance. The peak at 2.013 is ascribed to the formation of thiyl radicals under photoillumination.^19^ The sloped peak extended to higher g values indicates that holes are trapped at the S atoms.

**Figure 3:**
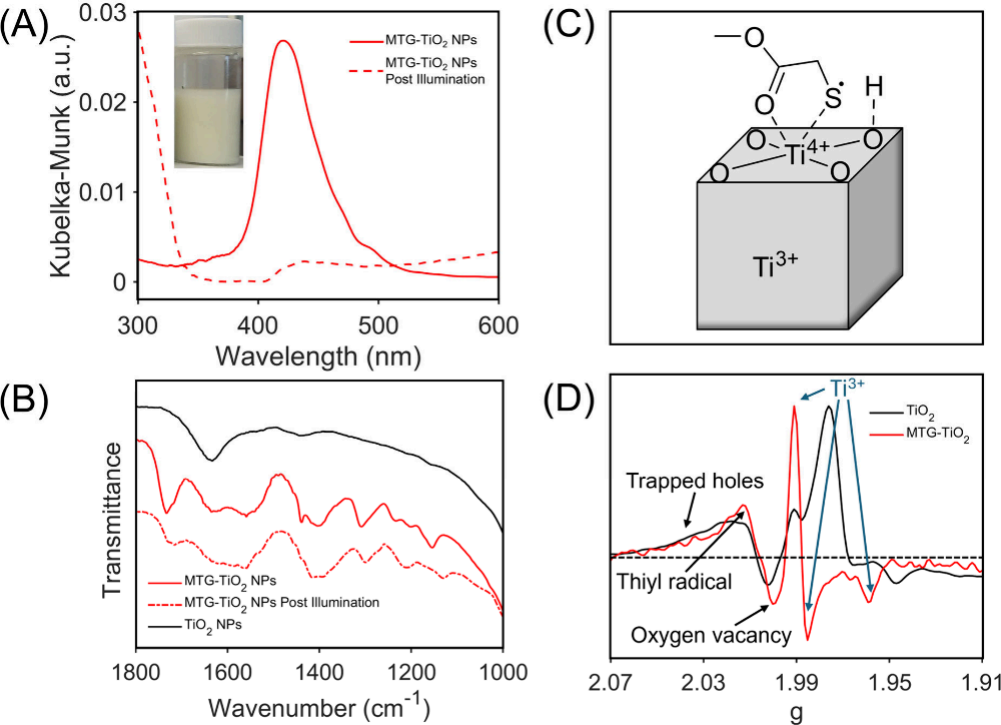
Spectroscopic characterization of the surface complex formed through absorption of MTG molecules on the P25 TiO_2_ nanoparticles. (A) UV–visible DRS spectra of MTG-modified nanoparticles before (solid curve) and after (dashed curve) illumination with LED light. The dashed curve intensity was increased by a factor of 5 for improved viewing clarity. The inset shows a photo of the dispersion of the MTG-modified nanoparticles, displaying a light-yellow color. (B) IR absorption spectra of the MTG-modified nanoparticles before (solid red curve) and after (dashed red curve) illumination with the LED light. The spectrum of the pristine P25 TiO_2_ nanoparticles is also plotted for reference (black solid curve). (C) Illustration of the chelate adsorption configuration of an MTG molecule on the TiO_2_ surface. (D) Cryo EPR spectra of the pristine P25 TiO_2_ nanoparticles (black curve) and the MTG-modified nanoparticles (red curve) at photoexcited states. The pristine P25 TiO_2_ nanoparticles were excited with a 400 nm laser, while the MTG-modified nanoparticles were excited with a Xe-arc lamp filtered through a water column and a 455 nm long-pass filter. Samples were held at 10 K for the duration of EPR experimentation.

Spectroscopic studies reveal the formation of thiyl radicals on TiO_2_ nanoparticle surfaces under illumination with visible light from an LED. It is known that thiyl radicals can react with phosphines to form phosphoranyl radicals, which are subsequently subject to β-scission fragmentation to give phosphine sulfides and reactive carbon radicals.^2^ When an MTG molecule is adsorbed to the TiO_2_ surface, the S and carbonyl O coordinate with surface Ti atom via a chelate configuration and H binds to surface O atom, leading to dissociation of the S–H bond (Figure 4A). Absorbing visible light excites the MTG-TiO_2_ CT-complex to drive charge separation/transfer and the sequential redox processes, forming a thiyl radical, an oxygen lattice vacancy, and a reduced Ti­(III) atom (Figure 4B). The thiyl radical adsorbed on the nanoparticle surface can be intercepted by TPP to form a phosphoranyl radical, which is primarily adsorbed on the nanoparticle surface through an interfacial O–Ti interaction (Figure 4C). The formation of the phosphoranyl radical significantly weakens the S–Ti interaction present in the adsorbed thiyl radical. The following β-scission fragmentation of the phosphoranyl radical releases triphenylphosphine sulfide (TPPS), leaving a carbon radical on the nanoparticle surface. Due to its electron deficiency, the radical carbon is prone to bind to the reduced Ti­(III), thereby stabilizing the radical on the nanoparticle surface (Figure 4D). The alkene bond of styrene can attack the radical carbon to add them together through the formation of a new C–C bond (Figure 4E). According to the severely hindered kinetics when employing methyl styrene (see examples in Figures 1 and 3f,g) and others reported for stabilizing reactive intermediates by TiO_2_ surfaces,^20–22^ it is likely that the new carbon radical still binds to the nanoparticle surface. When an additional MTG molecule is adsorbed to the nanoparticle surface, the dissociation of the S–H bond provides a hydrogen atom to neutralize the carbon radical, releasing the C–C coupling product of methyl 4-phenylbutyrate. The H atoms on the nanoparticle surface can also be a source to neutralize the radical. The photocatalytic reaction mechanism illustrated in Figure 4 is proposed as a promising pathway for reductive C–C coupling to aryl alkenes via desulfurization of MTG on TiO_2_ nanoparticles under visible light illumination.

**Figure 4:**
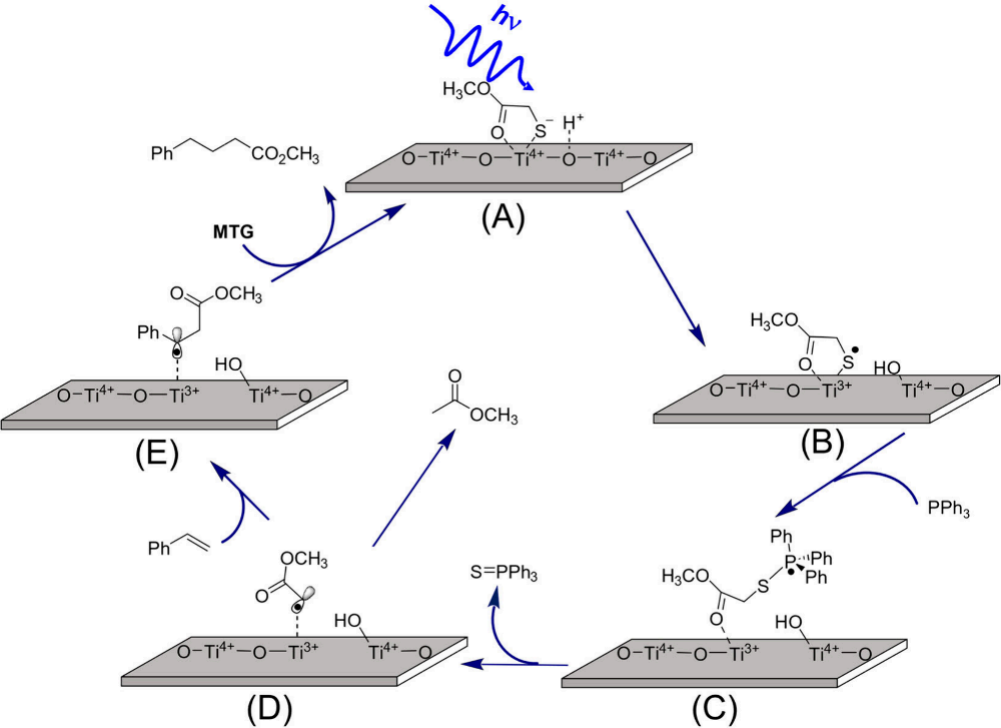
Illustration of the mechanism for photocatalytic C–C coupling to aryl alkenes via desulfurization of MTG by TPP on TiO_2_ nanoparticles under the illumination of visible light. In the absence of styrene, the carbon radical is neutralized by H to release desulfurization product, i.e., methyl acetate.

In summary, we established that TiO_2_ nanoparticles can function as visible-light photocatalysts for thiol desulfurization via a surface-mediated mechanism, despite the absence of intrinsic visible-light absorption by any individual reaction component. Adsorption of thiols on TiO_2_ induces the formation of a surface CT-complex that enables visible-light absorption and directly activates the bound thiol. Subsequent C–S bond cleavage generates carbon-centered radical intermediates, which can undergo the addition to aryl alkenes to afford C–C coupling products. The efficiency of this coupling is strongly influenced by radical steric demand with bulky carbon radicals suppressing C–C bond formation. In the absence of suitable alkene acceptors, the carbon radicals are quenched by hydrogen atom transfer to yield desulfurized products. More broadly, these findings demonstrate that engineering surface CT complexes offers a general strategy to impart visible-light activity to wide-bandgap metal oxides, enabling photocatalytic transformations under mild, cost-effective, and environmentally benign conditions using abundant TiO_2_ catalysts, ambient atmosphere, and room-temperature visible-light irradiation. The discovery of this work is promising to extend the scope of traditional photocatalytic transformation using semiconductor nanoparticle catalysts.^23–26^

## Supplementary Material



## Notes

Experimental details, C–C coupling with mixed styrenes, DRS spectra of TiO_2_ nanoparticles modified with different thiols, NMR spectra of washing solutions from MTG-modified TiO_2_ nanoparticles before and after photoillumination, and XANES spectra of MTG-modified TiO_2_ nanoparticles before and after photoillumination (PDF)
