# Supplementary material for: TiO2 Nanoparticles Enabling Photocatalytic Desulfurization for C–C Coupling Reaction Using Visible Light
Source: ACS Nanosci Au. 2026 Feb 27;6(3):363–8. doi: 10.1021/acsnanoscienceau.6c00020 (PMC13280997; doi:10.1021/acsnanoscienceau.6c00020)
Supplement: Supplementary file 1 [file ng6c00020_si_001.pdf]

Supporting Information

TiO_2 Nanoparticles Enabling Photocatalytic Desulfurization for C-C Coupling Reaction Using Visible Light

Shea Stewart[a], Matas Simukaitis[a], H. Christopher Fry[b], Chengjun Sun[c], Yugang Sun[a]*

[a] Department of Chemistry, Temple University, 1901 North 13th Street, Philadelphia, Pennsylvania 19122, USA

[b] Center for Nanoscale Materials, Argonne National Laboratory, 9700 South Cass Avenue, Lemont, Illinois 60439, USA

[c] X-ray Science Division, Advanced Photon Source, Argonne National Laboratory, 9700 South Cass Avenue, Lemont, Illinois 60439, USA

* Email: ygsun@temple.edu

- Experimental details
- Figure S1-S4

Experimental Details

1. Materials and Reagents

Aeroxide® P25 TiO_2 nanoparticles (a mixture of anatase phase and rutile phase) purchased from Acros Organics were used for all experiments unless stated otherwise. Other than the P25 nanoparticles, pure anatase-phase TiO_2 nanoparticles with an average size of 5 nm purchased from US Research Nanomaterials, Inc. were also used for comparison. Chemical reagents including styrene, thiol, and phosphine were purchased from Fisher Scientific and Sigma Aldrich, and used as received. Solvents were purchased from Fisher Scientific and used without further purification.

Reducing surfaces of the TiO_2 nanoparticles was performed through $NaBH_4$ treatment as reported in the literature.[1] The P25 TiO_2 nanoparticles and $NaBH_4$ powders were mixed at a molar ratio of 1.3:1 and ground using a mortar and pestle until thoroughly mixed before heating in a tube furnace set at 300 °C for 1 hour under an inert atmosphere of argon. The final blue powder was washed with plenty of water and then with ethanol before drying in an oven set at 60 °C overnight. The resulting sample was labeled as r- TiO_2 nanoparticles.

2. Photocatalytic Reaction and Analysis

A typical reaction was carried out in a 4-mL glass vial. The TiO_2 nanoparticles were weighed with an appropriate amount and added to the vial, followed by the addition of triphenylphosphine (TPP) and 2 mL of solvent (e.g., acetonitrile). After complete dissolution of TPP, thiol and styrene with appropriate amounts were then added to the vial. The vial was capped and placed into a house-made reaction holder possessing holes drilled into an aluminum block, which helps quickly dissipate heat generated from photothermal effect. The solution temperature was maintained around room temperature during photoreaction by blowing a fast stream of air onto the aluminum block. A white LED lamp (Dolan Jenner Fiber-Lite MI-LED B1) provided light with a power output of 0.75W/cm^2 and wavelength >420 nm. A typical reaction time was 24 hours although a shorter or longer time was also used for control experiment.

The reaction products were analyzed by sampling an aliquot of the reaction solution. The solution was subjected to centrifugation at 13,400 rpm for several minutes to settle down the nanoparticles at the bottom of the centrifuge tube. A 50-μL sample of the supernatant was added to 500 μL of deuterated chloroform ($CDCl_3$) containing 5 μL of cinnamaldehyde that serves as the

internal standard for quantification. The solution was then analyzed with ^{1}H NMR. The NMR spectra were obtained using a Bruker Advance 500 MHz spectrometer. Yields were determined by setting the integrated area of the cinnamaldehyde aldehyde proton to 1000 and using the fact that this signal corresponds to 0.0397 mmol of protons (via 5 μL of standard) to calculate amounts of analyte(s), accounting for signals corresponding to more than one proton.

If air was to be excluded from the reaction, the reaction solution was purged with argon via a balloon and needle whilst sonicating for 5 minutes. The outlet needle was then removed while leaving the argon balloon attached to maintain the inert atmosphere above the reaction solution during the entire reaction.

3. Characterization

UV-visible absorption spectra were recorded using a Thermo Scientific Evolution 220 spectrophotometer outfitted with an integrated sphere for measuring diffuse-reflectance spectra of dried powders. For measurement of charge-transfer (CT) complexes, a baseline measurement of the untreated TiO_2 nanoparticles were used rather than the standard background sample provided by the manufacturer. In this way, absorbances for dried nanoparticle powders modified with the reaction reagent(s) were measured. The surface modification was processed by stirring 100 mg of the P25 TiO_2 nanoparticles in 2 mL of acetonitrile (solvent) containing 0.6 mmol of each reagent for 4 hours. The nanoparticles were then collected via centrifugation at 13,400 rpm for several minutes, followed by drying in a vacuum for 15 minutes. The dry powder was then ground in a mortar and pestle until a nice flowing powder was obtained. The powder was then placed into the sample holder and measured. The sample was then photoilluminated for 15 minutes while still in the sample holder. The spectrum was then measured again to show the effect of photoillumination.

Infrared (IR) absorption spectra were taken on a Thermo Scientific Nicolet iS5 equipped with an iD5 ATR attachment. The samples for IR measurements were prepared in the same way as those for UV-visible absorption spectroscopy.

The electron paramagnetic resonance (EPR) spectroscopy studies were performed at the Center for Nanoscale Materials, a U.S. Department of Energy Office of Science User Facility. Measurements were made using an EPR EleXsys 500-E (CW) instrument with 2-mW power, modulation amplitude of 10, and microwave frequency of ~9.387 GHz. EPR measurements were taken from the powders of TiO_2 nanoparticles modified with methyl thioglycolate (MTG). The

sample of MTG-TiO_2 made from mixing 7 mg of anatase TiO_2 nanoparticles in 1 mL of acetonitrile with 3 μL of MTG. The mixture was stirred for a couple of hours before 200 μL of solutions was placed into a glass tube and immediately placed into liquid He. Samples were then mounted in the instrument and brought to 10 K, and this temperature was maintained for the duration of each experiment. Experiments consisted of taking measurements after a few minutes of illumination of the samples by a Xe-lamp while employing a series of long-pass filters. For example, one sample was illuminated for several minutes using a 500-nm long-pass filter and a measurement was made. The filter was then replaced with a 475-nm long-pass filter for another measurement. The similar operation was repeated till the use of a 400-nm long-pass filter. A column of water was used to filter out IR-emission from the Xe-lamp. For reference, the P25 TiO_2 nanoparticles were measured after band-gap excitation using a 400 nm laser.

X-ray diffraction (XRD) patterns were recorded on a Bruker D8 X-ray diffractometer with a Cu K_α (λ = 0.154 nm) source.

The samples for Ti K-edge X-ray absorption measurement were prepared similarly as those for those used for UV-Visible absorption spectroscopy. In preparation for measurement, the powders were sifted through a fine mesh then brushed onto the adhesive side of a strip of Kapton tape using a small paint brush for 20 minutes to create a uniform film of particles. The tape was then folded onto itself twice and fastened to a sample holder for measurement. After the initial measurements, the sample was unfurled, exposed to white-LED illumination (~0.75 W/cm^2) for 30 minutes, refolded and re-measured.

Titanium K-edge measurements were acquired at the 20-BM beamline of the Advanced Photon Source at Argonne National Laboratory. Measurements were taken in transmission mode with the beam intensity being monitored before and after passing through the sample via ionization chambers filled with N_2. The X-ray energy impingent upon the sample was controlled by stepping a Si(111) dual crystal monochromator operating at 15% detuning. The energy range measured was 4,816 ~ 5,936 eV corresponding to -150 and +970 eV relative to the E_0 of 4966 eV. The stepping program used was −150 to −20 (10 eV steps), −20 to +30 (0.3 eV steps) and +30 to +970 (0.05 eV steps) with an integration time of 0.5 seconds per step. Three scans were made for each sample.

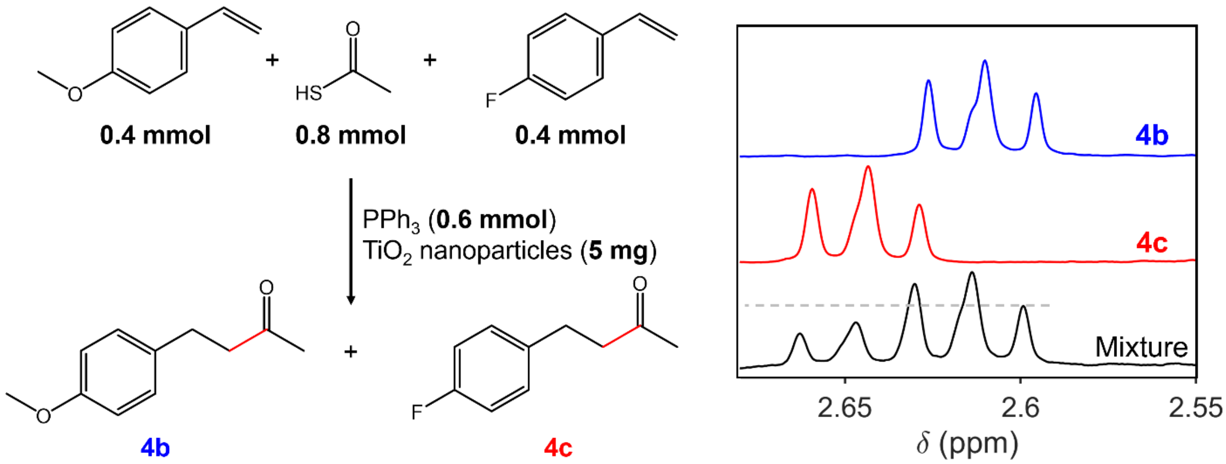

Figure S1. C-C coupling reaction from a solution containing two styrene derivatives with electronically opposed substituents, highlighting the apparent electrophilicity of the radicals derived from desulfurization of thioacetic acid.

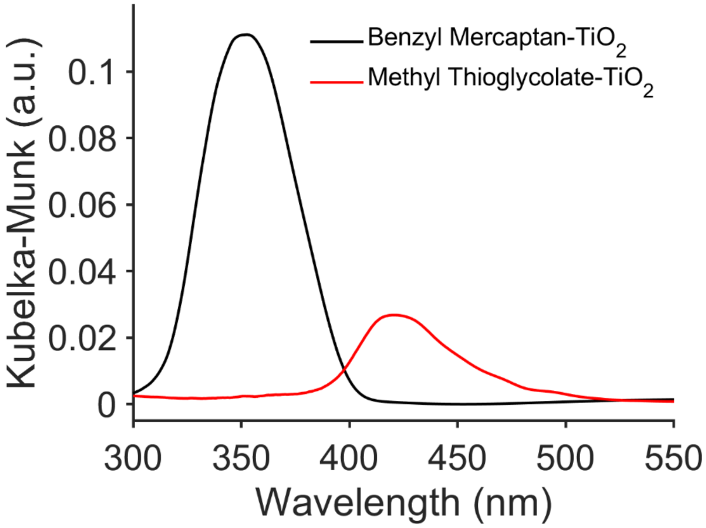

Figure S2. UV-visible DRS spectra of the P25 TiO_2 nanoparticles modified with benzyl mercaptan (black curve) and methyl thioglycolate (red curve).

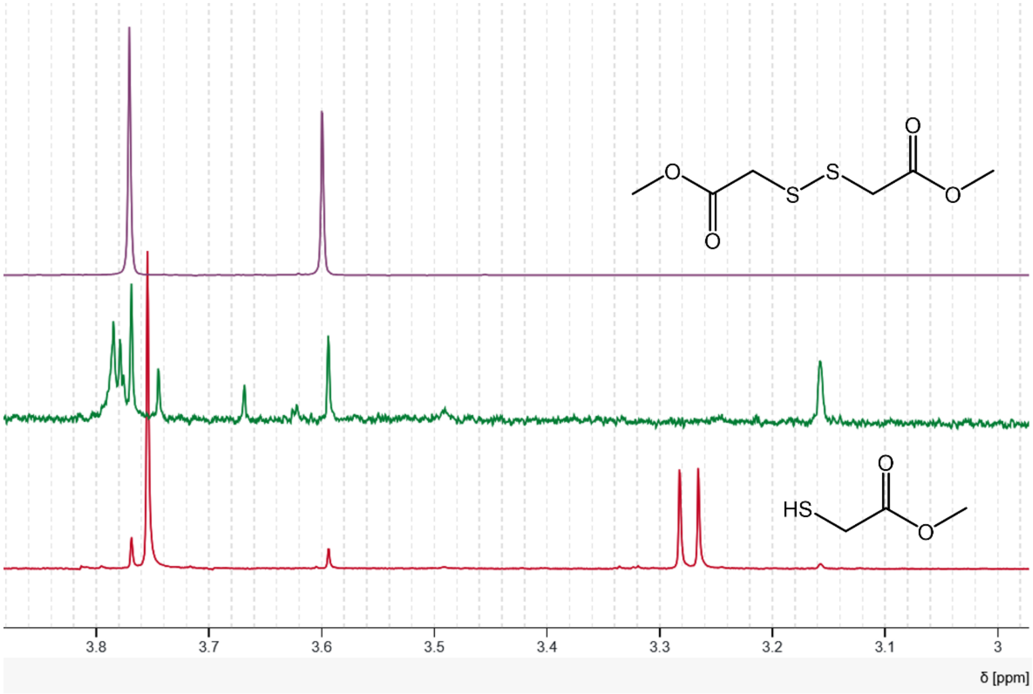

Figure S3. ^{1}H NMR of the rinse solutions collected from washing the MTG-TiO_2 nanoparticles with $CDCl_3$ before (red curve) and after (green curve) photoillumination. The peaks of the red curve are consistent with pure MTG molecules. The purple curve is the NMR spectrum of pure MTG disulfide in $CDCl_3$. The peaks of pure MTG disappear in the green curve, indicating the transformation of MTG to other species. Some peaks in the green curve align with the NMR spectrum of MTG disulfide, indicating that photoillumination transforms MTG to MTG disulfide. Other peaks might be ascribed to further photodegradation products consistent with previously reported literature.[2]

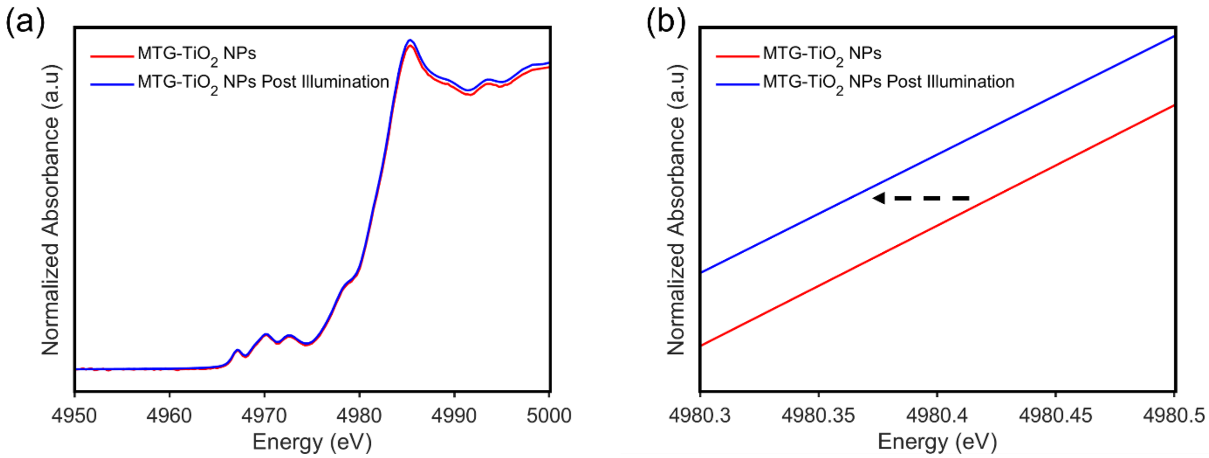

Figure S4. (A)XANES spectra and (B) the zoomed-in view of the white lines of Ti K-edge for the MTG-TiO_2 nanoparticles before (red) and after (blue) explicit illumination by a full-spectrum white LED. The absorption edge shifts to lower energy upon illumination, indicating the evolution of Ti^{4+} to Ti^{3+} during photoillumination.

(1) Wang, J.; Peng, Y.; Wang, L.; Yang, X.; Gao, Y.; Li, X.; Lü, W. Modification of TiO_2 with sodium borohydride and its influence on photovoltaic cell performance. *Ionics* **2024**, *30* (7), 4271–4279. https://doi.org/10.1007/s11581-024-05551-z.

(2) Seng, S.; Picone, A. L.; Bava, Y. B.; Juncal, L. C.; Moreau, M.; Ciuraru, R.; George, C.; Romano, R. M.; Sobanska, S.; Tobon, Y. A. Photodegradation of methyl thioglycolate particles as a proxy for organosulphur containing droplets. *Phys. Chem. Chem. Phys.* **2018**, *20* (29), 19416–19423. https://doi.org/10.1039/C7CP08658J.
